# Fatal Cases of Influenza A in Childhood

**DOI:** 10.1371/journal.pone.0007671

**Published:** 2009-10-30

**Authors:** Benjamin F. Johnson, Louise E. Wilson, Joanna Ellis, Alex J. Elliot, Wendy S. Barclay, Richard G. Pebody, Jim McMenamin, Douglas M. Fleming, Maria C. Zambon

**Affiliations:** 1 Centre for Infections, Health Protection Agency, London, United Kingdom; 2 Department of Virology, Faculty of Medicine, Imperial College London, London, United Kingdom; 3 Health Protection Scotland, Glasgow, United Kingdom; 4 Royal College of General Practitioners Research and Surveillance Centre, Birmingham, United Kingdom; Columbia University, United States of America

## Abstract

**Background:**

In the northern hemisphere winter of 2003–04 antigenic variant strains (A/Fujian/411/02 –like) of influenza A H3N2 emerged. Circulation of these strains in the UK was accompanied by an unusually high number of laboratory confirmed influenza associated fatalities in children. This study was carried out to better understand risk factors associated with fatal cases of influenza in children.

**Methodology/Principal Findings:**

Case histories, autopsy reports and death registration certificates for seventeen fatal cases of laboratory confirmed influenza in children were analyzed. None had a recognized pre-existing risk factor for severe influenza and none had been vaccinated. Three cases had evidence of significant bacterial co-infection. Influenza strains recovered from fatal cases were antigenically similar to those circulating in the community. A comparison of protective antibody titres in age stratified cohort sera taken before and after winter 2003–04 showed that young children had the highest attack rate during this season (21% difference, 95% confidence interval from 0.09 to 0.33, p = 0.0009). Clinical incidences of influenza-like illness (ILI) in young age groups were shown to be highest only in the years when novel antigenic drift variants emerged.

**Conclusions/Significance:**

This work presents a rare insight into fatal influenza H3N2 in healthy children. It confirms that circulating seasonal influenza A H3N2 strains can cause severe disease and death in children in the apparent absence of associated bacterial infection or predisposing risk factors. This adds to the body of evidence demonstrating the burden of severe illness due to seasonal influenza A in childhood.

## Introduction

Influenza A virus is a cause of severe morbidity and mortality worldwide [1], [2]. In the developed world, most influenza-associated deaths occur in the elderly but fatal outcomes occur regularly in young adults and children [3], [4].

Human seasonal influenza A viruses evolve rapidly through genetic mutation, allowing them to escape previously acquired immunity [5]. Viral variants that are altered in their antigenic properties emerge frequently, a phenomenon described as antigenic drift, which is particularly marked with human influenza A H3N2. A/Fujian/411/02-like influenza A H3N2, first noted in sporadic cases of returning U.K. travellers in early summer 2003 [6] was antigenically distinguishable from the previously circulating H3N2 drift variant, A/Panama/2007/99 and replaced it to became the only circulating strain in winter season 2003–04 [7].

This study investigated fatal cases of influenza H3N2 in children during the emergence of A/Fujian/411/02 in the UK in winter 2003–04. The goals of the study were: to investigate whether any of the case fatalities had underlying disease or risk factors; to evaluate how laboratory confirmed infections were recorded in death certification; to describe the clinical and pathological findings in relation to circulating virus strains; and to investigate whether the high number of laboratory confirmed fatalities was reflected in measures of morbidity in primary care, age specific attack rates and age specific all-cause mortality. We reviewed the antecedent illness history, underlying risk factors, clinical findings and observed pathology at autopsy. Age-stratified serological data were combined with community based clinical morbidity data to provide an assessment of the overall population immune susceptibility, attack rates and impact of A/Fujian/411/02-like influenza in comparison with other winter seasons in which new H3N2 antigenic variants emerged.

## Materials and Methods

### Ethics statement

This study was conducted as part of a public health inquiry by the Health Protection Agency and Health Protection Scotland into paediatric deaths, where it was agreed that ethical approval for linking personal clinical outcome information to virus strains was not required. Nationally representative, anonymised, age stratified residual sera were obtained from the HPA serum bank held in Preston NHS hospital. Ethical approval was given by the HPA for the collection of these samples.

### Case definition and confirmation of influenza virus infection

A case was defined as a death in child less than eighteen years of age during the 2003–04 influenza season (1^st^ September 2003 to 30^th^ May 2004) with laboratory confirmation of influenza virus infection reported to HPA. Most specimens were initially tested at referring hospitals, and all were subsequently tested at the national influenza laboratory, HPA Centre for Infection, Colindale (HPA CfI). Microbiological findings deemed significant are included.

### Demographic and Clinical Characteristics

Health care providers contacted local public health authorities and HPA CfI to report cases. Available clinical records and data, autopsy reports and microbiology results were obtained as part of the public health investigation for all cases and were reviewed by a senior paediatrician and other specialist medical staff (all of whom are authors on this paper). The activity took place within the context of a national public health enquiry into influenza associated childhood deaths in winter 2003. Children without risk factors recognized by UK Joint Committee on Vaccination and Immunisation to increase the risk of influenza related complications were classified as “previously healthy”. U.K. risk factors for severe influenza from 2003–04 are given in reference 12. Verification of the certified cause of death in each fatal case was performed by matching cases to Office of National Statistics (ONS) death registration records held at HPA.

### Surveillance data, clinical incidence data for influenza-like illness (ILI) and death registration data

The Weekly Returns Service (WRS) of the Royal College of General Practitioners is a clinical information system based upon a network of sentinel general practices located throughout England and Wales [8]. Mean weekly incidence rates per 100,000 population for first and new episodes of ILI (ICD9 487) were calculated for “influenza virus active weeks” during each winter (weeks 40 to 20) as previously described [8]. Data were aggregated into the age groups; all-age, 0–4, 5–14, 15–44, 45–64 and 65+ years. Surveillance data about circulating influenza viruses in the U.K. is recorded weekly in the HPA National Influenza Report, available online at http://www.hpa.nhs.uk/web/HPAweb&HPAwebStandard/HPAweb_C/1195733836222.

### Cells and viruses

Viruses were grown in either MDCK or MDCK-SIAT1 cells and subtyping was performed by RT-PCR, if no virus isolate was available [9]. Virus isolates were characterized antigenically using post infection ferret antisera using haemagglutination inhibition (HI) assays [10].

### Age Stratified Sera

Nationally representative, anonymised, age stratified residual sera were obtained with ethical approval from the HPA serum bank held in Preston NHS hospital. The number of sera tested from 2003 was 88 (ages 0–4), 196 (ages 5–17), 293 (ages 18–44), 185 (ages 45–64) and 101 (ages 65+). The number of sera from 2004 for each age group was 70 (ages 0–4), 160 (ages 5–17), 273 (ages 18–44), 199 (ages 45–64) and 98 (ages 65+).

### Haemagglutination Inhibition Assay

Haemagglutination inhibition (HI) assays to assess protective antibody titres in human sera were performed using standard protocols and antigenically representative viruses. A titre of >1/40 was taken as protective.[11] The proportion of sera in 2003 and 2004 with protective antibody titre were compared using a 2-sided Fisher's exact P (p values are indicated).

## Results

### Clinical Findings

Seventeen fatal cases of laboratory-confirmed influenza A in children under eighteen years were reported to HPA between September and December 2003. This period corresponded with peak circulation of influenza during the winter 2003–04. The median age of fatal cases was two years, with a range from four months to seventeen years; the majority (64%) were under five years. Ten (58%) deaths were in females. The duration of illness prior to death varied, with a median duration of three days (range 0–14 days). Four (24%) deaths were sudden with either no recognized antecedent illness or illness of less than 6 hours duration. A further four (24%) occurred within forty-eight hours of onset of symptoms, five (29%) were ill for three to four days prior to death, three (18%) were ill for a week prior to death, and one individual was unwell for over ten days. Eight (47%) children died following hospitalization and nine (53%) died in the community. In the cases with recognized antecedent illness, eight (57%) had fever, eleven (79%) had respiratory symptoms (cough/chest infection/croup), three (21%) had vomiting and/or diarrhea, two (14%) had clinically recognized sepsis. Two (14%) children were markedly confused prior to collapse (Table 1).

**Table 1:** Summary of fatal paediatric cases of influenza H3N2 from 2003–04.

Case No.	Time between illness onset and death	Clinical illness	Post Mortem Findings
1	7 days	Flu like symptoms for several days with vomiting and diarrhoea.	Congested larynx and trachea. No focal consolidation. Enlarged liver.
2	Sudden Death	Few hours temperature, vomiting, confusion and rapid collapse. Cerebral oedema CT scan.	Patchy intraalveolar haemorrhage. Hypoxia & swollen brain. Norovirus detected.
3	3–4 days	Vomiting over several days. Rapid collapse and terminal deterioration.	Acute bronchitis and early bronchopneumonia. Intraalveolar haemorrhage. *Staphlococcus aureus* septicaemia.
4	1–2	Mild respiratory illness. Sudden death.	Diffuse alveolar damage and intraalveolar haemorrhage. Tracheobronchitis. Enterovirus detected
5	7 days	Mild illness. Hypothermia and circulatory collapse, terminal deterioration.	Widespread myocarditis. Tracheobronchitis. Pulmonary oedema. Pleural effusion.
6	14 days	Fever, cough and chest infection. Cardiac arrest.	Pulmonary oedema Bilateral pulmonary & pericardial effusion. Diffuse widespread myocarditis. “Starry sky” and haemorrhagic lymph nodes with lymphadenopathy.
7	1 day	Sore throat and temperature. Sudden collapse.	Tracheitis and Epiglottitis. Lung congestion Peribronchial infiltrate. Lymphadenopathy, haemorrhagic spleen. Acute hypoxia/ischaemia in the brain
8	Sudden Death	No.	Bronchopneumonia
9	4 days	Ear infection. Fever, cough and cold. Collapse.	Tracheal ulceration and epiglottitis. Intraalveolar haemorrhage Lung infiltrates. Haemorrhagic spleen
10	Sudden Death	No.	Tracheitis. Intrapulmonary haemorrhage. Enlarged, haemorrhagic thymus
11	Sudden Death	No.	Tracheitis. Intraalveolar haemorrhage. Unusual laryngeal musculature.
12	3 days	Cough for few days. Apnoea.	Bilateral pleural effusions. Pulmonary oedema. Severe ulcerative tracheitis. Lymphadenopathy
13	2 days	Fever and tachypnoea.	Congested lungs. Severe ulcerative tracheitis. Starry sky lymph nodes. Haemorrhagic thymus. Enlarged spleen.
14	3 days	Croup like illness 2–3 days.	Tracheobronchitis. Interstitial haemorrhage. *Streptococcus pneumoniae* septicaemia.
15	7 days	Flu like illness and lethargy.	Bronchopneumonia with intraalveolar haemorrhage, necrosis and secondary. *Streptococcal* pneumonia. Lymphadenopathy
16	3 days	Mild respiratory illness. Lethargy then haemopytsis. Chest X-ray consolidation	Haemorrhagic pneumonia.
17	1 day	High temperature and lethargy.	Brain oedema and lung collapse. Lymphadenopathy and haemorrhagic lymph nodes.

Among the sixteen cases for whom underlying health status information was available none was recorded as having a known risk factor for severe influenza, although two cases (both over two years) had a history of febrile convulsions [12]. No child received influenza vaccine for winter 2003–04.

In nine cases (53%) influenza A detection occurred by more than one method (RT-PCR, DIF or virus isolation), either in respiratory clinical samples or from post-mortem tissues. In eleven (64%) cases influenza A virus isolates were recovered. The cultured viruses were analyzed for their antigenic characteristics; all isolates were found to be antigenically similar to each other and to virus isolates recovered from non-fatal cases matched for age, week of infection and geographical location (controls) as well as to other circulating strains from the same locality (data not shown). Influenza AH3N2 was detected by RT-PCR only in five (29%) cases.

*Staphylococcus aureus* and *Streptococcus pneumoniae* in blood were identified in the two cases of clinical sepsis and one case had *Streptococcus pneumonia* bacterial pneumonia. No other significant bacteriological findings were described. Enterovirus and norovirus were also detected by RT-PCR in two cases.

Autopsy reports were available for all cases (Table 1). All cases showed involvement of the airways including congestion, epiglottitis, ulcerative tracheitis, bronchitis, tracheobronchitis bronchopneumonia, pneumonitis, unilateral or bilateral pneumonia. Lung pathology included congestion, intraalveolar haemorrhage, necrosis, diffuse alveolar damage, pulmonary edema, lung infiltrates, pleural effusions, hemorrhagic pneumonia and lung collapse. Extra-pulmonary pathology included two cases (12%) with marked cerebral edema suggestive of hypoxia, sepsis in two cases (12%) due to *Staphylococcus aureus* or *Streptococcus pneumoniae* and myocarditis in two cases (12%). Eight cases (47%) showed abnormalities of the lymphoid system, including seven (41%) with enlargement of the lymph nodes (LN), thymus or spleen, six (35%) with hemorrhagic LN, thymus or spleen and two (12%) with “starry sky” LN indicating an infiltration of macrophages in the lymphoid tissue. No metabolic disorders were recognized ante or post mortem, although one case showed unusual large fiber laryngeal musculature of uncertain significance.

### Death registration

Death registration information was not available for one case. By routine death registration only nine of sixteen fatal cases (56%) were coded with influenza (ICD10 J10–J11). Seven of these specifically mentioned influenza A on the death certificate and a further certificate mentioned “influenza” when text was searched manually. Two (13%) of the deaths were registered as pneumonia (J18); two (13%) as other respiratory disorders (J98); two (13%) were registered as sudden infant death (SIDS) and one as acute myocarditis, without mention of influenza (I40). Septicemia (A41) and myocarditis (I40, I514) were identified as contributing causes to death on three certificates. No death certificate indicated any chronic medical conditions known to increase the risk of severe disease from influenza infection.

### Analysis of the age related susceptibility to influenza A H3N2 infection

**Figure 1:**
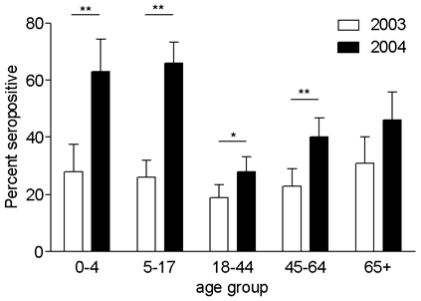
Age-distribution of seroconversion to influenza virus in 2003 and 2004. Charts show the proportion of sera from the summers of 2003 (clear bars) and 2004 (filled bars) that gave a HI titre above 1/40 for each age group against A/Wyoming/3/03 (antigenically equivalent to A/Fujian/411/02), together with the 95% confidence interval (error bars). Asterisks denote statistical significance (*p<0·05; **p<0·01). A total of 882 and 799 serum samples were tested for 2003 and 2004 respectively, each in duplicate, together with control ferret sera.

We sought to establish whether young children showed particular susceptibility to A/Fujian/411/02-like virus infection during this winter season by analyzing sera from summers 2003 and 2004, prior and subsequent to the emergence of this antigenic drift variant. Serum samples in a nationally representative age-stratified serum cohort were analyzed for the presence of protective antibodies (figure 1). The number of individuals (all ages) with a HAI titre >1/40 increased from 207/863 (24%) in 2003 to 348/800 (34%) in 2004 (p<0.0001). The largest increase was seen in children under the age of four years, where the number of individuals with a titre >1/40 rose from 25/88 (28%) in 2003 to 44/70 (63%) in 2004 (p<0.01). This compares to 304/730 (42%) of individuals over 4 years with a HAI titre >1/40 in 2004 (21% difference, 95% confidence interval from 0.09 to 0.33, p = 0.0009). This shows that those aged less than 4 years in 2004 were significantly more likely to have been infected with the novel strain A/Fujian/411/02-like influenza during winter 2003–04.

### Population Morbidity Estimates

**Figure 2:**
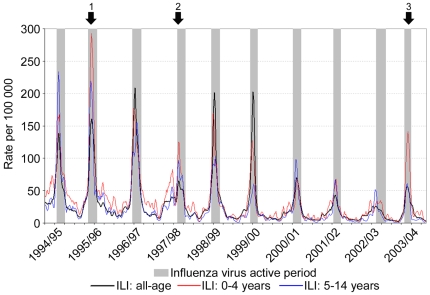
Clinical incidence rate of influenza-like illness (ILI) per 100,000 population recorded by the Royal College of General Practitioners Weekly Returns Service. Weekly incidence rate of ILI presented for all-age, 0–4 and 5–14 years age groups. Vertical shaded bars represent defined periods of influenza virus circulation derived from laboratory reports.[8] Arrows indicate those years in which a new variant was introduced into the community (1. A/Wuhan/359/95; 2. A/Sydney/5/97; 3. A/Fujian/411/02).

**Figure 3:**
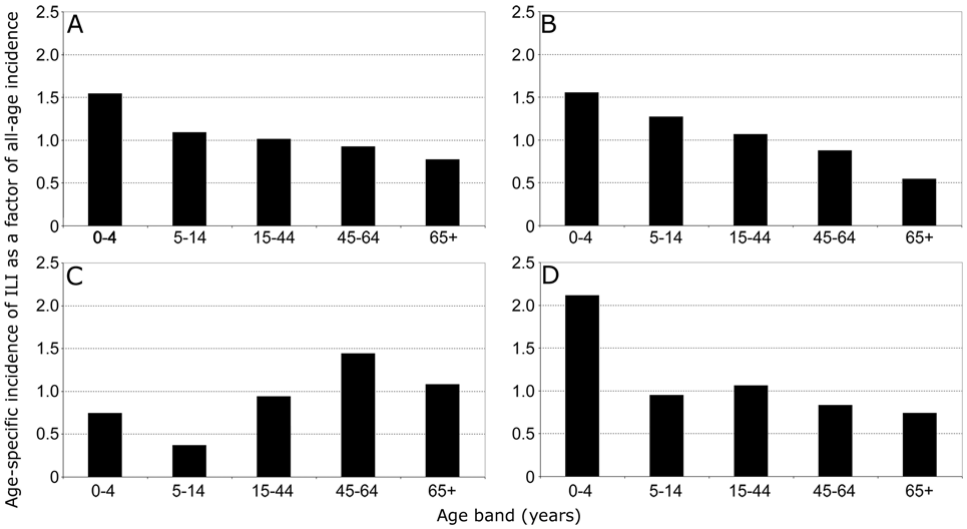
Clinical incidence rate of influenza-like illness (ILI) by age group expressed as a factor of the all-age incidence rate. Mean weekly incidence rates of ILI during influenza virus active weeks were calculated for winters: A) 1995/96 (A/Wuhan/359/95); B) 1997/98 (A/Sydney/5/97); C) 1999/00 (no new variant); and D) 2003/04 (A/Fujian/411/02).

To assess the morbidity of influenza in different age groups we analysed observational clinical data from primary care recorded by GPs of the Weekly Returns Service (WRS). This is a national sentinel network which has been recording influenza-like illness (ILI) for over 40 years, and for which linked clinical-virological surveillance has been operational since 1994. The all-age incidences of ILI in England and Wales over a ten year period from 1994–2004, together with incidence rates in children ages 0–4 and 5–14 years is shown in Figure 2. During the ten year period, there were only three seasons where the incidence of morbidity in children aged 0–4 years exceeded the all-age incidences (marked by arrows). All three seasons corresponded to the emergence of significant antigenic drift variants of influenza A H3N2 in 1995–96 (A/Wuhan/359/95), 1997–98 (A/Sydney/5/97) and 2003–04 (A/Fujian/411/02). During this period of time there were no other new H3N2 drift variants which emerged and dominated virus circulation. The mean weekly incidence rates of ILI were compared for different age groups during these influenza seasons, together with 1999–2000 as an example of a winter season with a dominance of circulating influenza A H3N2 and high population morbidity (Figure 2), but where a new drift variant did not emerge. The emergence of significant antigenic drift variants in 1995–96, 1997–98 and 2003–04 was accompanied by the highest incidences of ILI in those aged 0–4 years (Figure 3A, B and D). The season 2003–04 was notable for the fact that population morbidity rates in young children aged 0–4 were more than twice those of the overall all-age morbidity, indicating substantial disease burden in the young. This is consistent with the serological findings of the highest age specific attack rates having occurred in the youngest age groups during this winter period. In the winter 1999–00, where a new antigenic drift variant did not emerge, the highest rates of ILI morbidity rates were recorded in the older age groups 45–64 and 65 and over (Figure 3C).

## Discussion

Winter 2003–04 was marked by an unusually high number of severe and fatal cases of influenza in the young, following the emergence of the influenza A H3N2 antigenic drift variant A/Fujian/411/02 [6]. The cases all occurred in children without known risk factors and for whom no vaccination would be recommended. The greatest susceptibility to the drift variant was in the youngest age group as demonstrated by age specific attack rates estimated serologically. This conclusion correlated with a separate determination of population morbidity recorded in primary care during the peak periods of virus circulation during the 2003–04 winter period, although there was no increase in all-cause mortality in children <14 years that year (HPA unpublished observations). Over a ten year period, years that had the highest population morbidity in the youngest age groups were those that saw the emergence and circulation of novel H3N2 antigenic drift variants. Together, this suggests that the most vulnerable individuals are those with the least immunity to influenza as a result of limited prior exposure to circulating strains, and we suggest that a gradual build up of immunity as a result of exposure correlates with protection in the face of a novel drift variant. Therefore, the emergence of drift variants or pandemic influenza strains will cause higher morbidity and mortality rates in young children due to the lack of cross reactive immunity, either cellular or humoral, raised during a previous natural infection. It is interesting to note that in a recent study of the current outbreak of novel swine-origin influenza A (H1N1), 60% of patients were 18 years of age or younger [13].

The range of clinical complications in UK fatal cases can be grouped into categories including (1) fulminant progression to death after an initially mild illness, (2) invasive bacterial infection, (3) respiratory tract complications and (4) non respiratory complications including myocarditis and encephalopathy. Extrapulmonary complications of myocarditis and encephalopathy were not accompanied by the detection of viral RNA from brain or heart tissue by RT-PCR, although virus was recovered from the nasopharynx. The pathological conclusions rest on histology findings, which suggests that tissue damage may occur through mechanisms other than direct viral replication, as has been previously suggested [14].

The clinical microbiological findings add to the debate about the importance of bacterial co-pathogens in influenza associated fatality. Only three (18%) of the fatal cases showed evidence of significant bacterial co-infection. This is similar to findings from fatal cases in the US 2003–04 and also to preliminary information from the current outbreak of swine-origin influenza A H1N1, where a minority of paediatric hospital admissions had possible or probable bacterial infection [3], [15]. This is substantially less than the figures for the 1918 (96%) or 1957 (75%) influenza pandemics [14], [16] or during the 2008–09 influenza season in the USA, where six (66.7%) out of nine reported deaths in children had bacterial coinfections, mainly *Staphylococcus aureus* [17]. It is possible that treatment with antibiotics in 2003–04 may have masked the contribution of bacterial pathogens to pathology, or that the post mortem bacteriological findings have been underestimated, although at least half of the fatal cases died without any therapeutics. Disparities in the assessment of contribution played by bacterial co-pathogens may reflect differences between adult and child fatal case series, and may also be due to variations between different strains of influenza.

In this case series over 40% of death certificates had no mention of influenza as a direct or indirect cause of death, and in over 70% of cases the diagnosis of influenza was not made until post mortem tissue was examined. The burden of influenza in young children is therefore under recognized, precisely because few influenza infections are recognized clinically [4]. Of the cases reported to HPA and Health Protection Scotland during 2003–2004, seventeen were laboratory confirmed for A/Fujian/411/02-like influenza. This number is not comprehensive and is likely to underestimate the number of fatal cases that occurred. Recognition of influenza can provide the opportunity for improved infection control, vaccination and antiviral therapy. Use of national mortality registration data to estimate deaths due to influenza in childhood will seriously underestimate the impact of influenza even if all cause mortality is considered.

A risk-factor based influenza vaccination program for children would not prevent these fatal cases as the reasons underlying susceptibility to severe disease remain cryptic. Further studies on the outcome of seasonal influenza in children will help us to predict the impact of future epidemics and will assist understanding of the outcome of infections in the immune naïve host during influenza pandemics.
